# Factors influencing caregiver burden by dementia severity based on an online database from Seoul dementia management project in Korea

**DOI:** 10.1186/s12877-021-02613-z

**Published:** 2021-11-19

**Authors:** Boyoung Kim, Jennifer Ivy Kim, Hae Ri Na, Kang Sook Lee, Kyung-hee Chae, Sukil Kim

**Affiliations:** 1 Department of Public Health, Graduate School, The Catholic University of Korea, Seoul, South Korea; 2 Department of Neurology, The Bobath Memorial Hospital, Seongnam-si, South Korea; 3 Department of Preventive Medicine, College of Medicine, The Catholic University of Korea, Seoul, South Korea

**Keywords:** Caregiver burden, Patients with dementia, Cognitive impairment, Clinical dementia rating

## Abstract

**Backgrounds:**

As the prevalence of dementia rises, caregiver burden also increases in South Korea, especially for informal family caregivers. This study aimed to analyze factors affecting caregiver burden by the severity of dementia based on data of patients in Seoul.

**Methods:**

A total of 12,292 individuals aged ≥65 years enrolled in the Seoul Dementia Management Project from 2010 to 2016 in an online database were selected. Caregiver’s burden was assessed using the Korea version of Zarit Burden Interview. Multiple regression analyses were performed to determine factors associated with primary caregiver’s burden after stratifying the severity of dementia.

**Results:**

Most patients showed moderate levels of cognitive impairment (49.4%), behavior problems (82.6%), and ADL dependency (73.6%). After stratifying the severity of dementia, caregivers caring for patients with mild symptoms of dementia were experienced with higher caregiver burden if patients were under a lower score of IADL. Significant factors for caregiver burden among caregivers supporting patients with moderate symptoms of dementia include caregivers’ residence with patients, subjective health status, and co-work with secondary caregivers. Lastly, caregivers for patients with severe dementia symptoms experienced a higher caregiver burden from limited cognitive function, problematic behavior, and caregivers’ negative health status.

**Conclusion:**

In terms of sample size, this study had far more patients than any other domestic or international study. It was meaningful in that it analyzed characteristics of patients with dementia and caregivers affecting the burden of caregivers in Korea. Intensive social supports with multiple coping strategies focusing on different levels of patients’ clinical symptoms and caregivers’ needs should be planned to relieve the caregiver burden.

## Background

A rapidly aging society affects not only socioeconomic terms, but also puts a heavy burden of caring for patients with dementia. In South Korea, the prevalence of dementia has gradually increased. It is expected to increase from 10.16% in 2018 to 16.4% in 2050 [1] due to the high speed of population aging. The first National Dementia Management plan and the long-term care insurance for the elderly in South Korea were started in 2008. After the Dementia Management Law enacted in 2012, the National Institute of Dementia (NID) and Dementia Care Advising Service in public health center has launched a suitable healthcare service for the elderly. Since then, 12 cities and provinces nationwide have set up and operated regional a dementia center. Following national plans for patients with dementia, 256 local governments set up Dementia Safe Centers at provincial levels in 2018, with governmental financial support to prevent worsening symptoms of dementia.

Although there are some national care plans for improving the health status of patients with dementia, there is relatively little support for caregivers of patients with dementia. Distress from caregiver reflects multidimensional responses of physical, emotional, and financial difficulty related to patients’ cognitive impairment, behavioral disturbance, and limited daily activities [2]. Although several studies have mentioned the significant impact of dementia on caregiver burden, significant determinants are inconsistent. Among patient variables, psychiatric symptoms have a substantial effect on caregiver burden [3]. In some studies, behavioral problems are the most influential factors in deciding the caregiver burden [4, 5]. In cases of caregiver determinants, caregivers’ neuroticism also has an influence on caregiver burden [6, 7]. However, in contrast with this result, other studies have revealed that caregiver’s gender [8] and self-efficacy [9] decide caregiver burden.

It has been reported that caregivers for patients with dementia experience several mental health problems such as anxiety and depression [10–13]. As the prevalence of dementia rises, the burden of caregiving also increases in South Korea, especially among informal family caregivers [14]. In Korea, a filial duty under Confucian background and a lack of skilled nursing facilities put families no choice but to become informal caregivers. Although family caregivers perform their roles in taking care of patients with a sense of duty and cultural norms, they are highly likely to show degraded quality of life and suffer psychological distress. Therefore, it is important to analyze factors associated with patients with dementia that may influence the caregiver burden [15, 16]. Thus, the aim of this study was to examine effects of characteristics of patients with dementia and their caregivers on caregiver burden in Korea.

## Methods

### Study settings

Seoul Dementia Management Project (SDMP) provides comprehensive healthcare programs under the supervision of Seoul Metropolitan Center for Dementia (SMCD) [17]. The SDMP emphasizes community-based integrative management for dementia, which encompasses education, preventive programs, early detection, therapeutic interventions, and proper care services that are closely linked to online case-registration & management systems. The online database from SDMP provides socio-demographic characteristics of patients, results of screening examination, and history of care programs such as detailed information of services to prevent worsening dementia symptoms, improve patients’ cognitive function, and provide social support for patients’ families.

### Participants

**Fig. 1:**
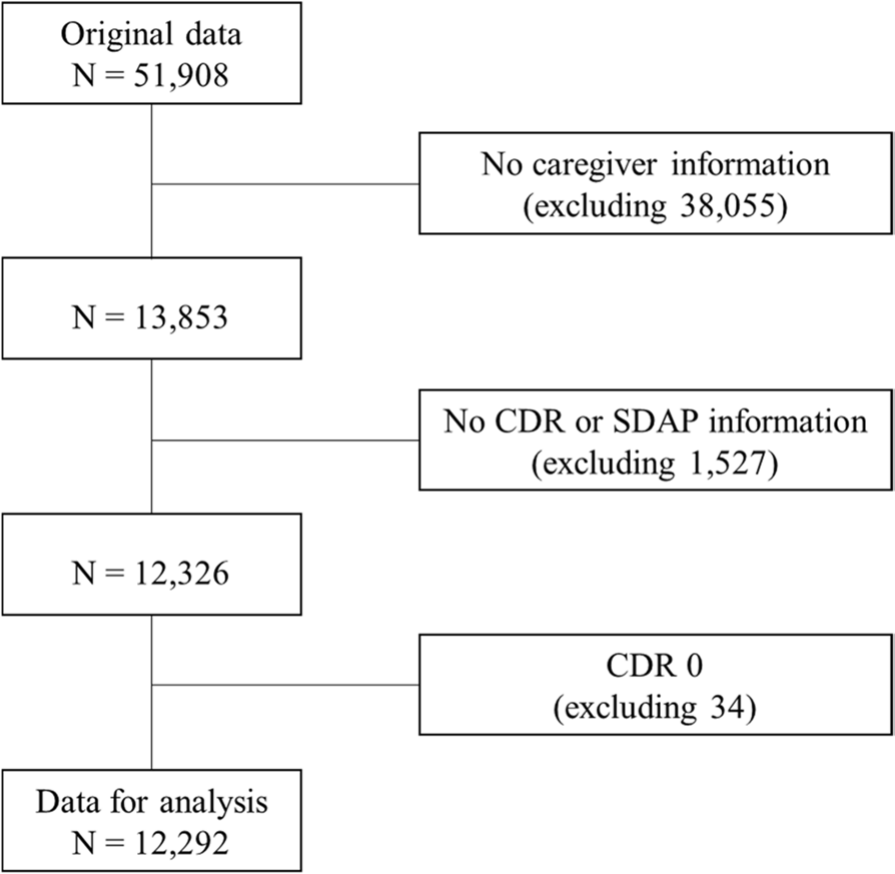
Flow chart showing the selection of samples for this study. Note. § CDR = Clinical Dementia Rating, SDAP = Seoul Dementia Assessment Packet

The Clinical Dementia Rating (CDR) scale, a generally used dementia staging instrument, was used by a panel of neurologists and psychiatrists from 25 districts of Dementia Safe Centers. CDR score was assessed using a collected clinical instrument during phase 1 and phase 2. This study followed global CDR criteria, a 5-point ordinal scale. CDR scores of 0.5 ~ 1, 2, and 3 refer to mild, moderate, and severe dementia, respectively, while CDR 0 indicates no dementia. In phase 1, a trained nurse performed a mental status examination to identify cognitive impairment. The cognitive impairment group had completed either Consortium to Establish a Registry for Alzheimer’s Disease or Seoul Neuropsychological Screening Battery [18]. In phase 2, neurologists and psychiatrists diagnosed patients’ symptoms under the basis of clinical assessment or Diagnostic and Statistical Manual of Mental Disorders-IV. Therefore, a total of 12,292 patients were analyzed after excluding those who had no information about their major caregiver (38,055), no Clinical Dementia Rating (CDR) information (1527), and patients with no dementia (CDR = 0) [19] from 51,908 patients from 2010 to 2016 in an online database (Fig. 1).

### Severity of dementia

The severity of dementia was assessed with the Seoul Dementia Assessment Packet (SDAP), a brief screening instrument that was developed to screen patients’ symptoms and caregiver burden in multidimensional aspects. SDAP was used to evaluate patients’ symptoms based on the summary score of cognitive impairment, Behavioral And Psychological Symptoms In Dementia (BPSD), Activities of Daily Living (ADL), and Instrumental Activities of Daily Living (IADL) [20].

Tools used for assessing the functional ability of patients with dementia are as follows. Cognitive impairment was measured by four items: memory, orientation, problem-solving, and communication skill. The severity of cognitive impairment was categorized into three levels based on total scores from the questionnaire: mild (0–4), moderate [5–8], and severe [9–12]. The severity of BPSD included six domains: delusion, hallucination, agitation, apathy, irritability, aberrant motor behavior, and sleep disturbance. The severity of BPSD was classified into three categories after summing scores from each questionnaire: mild (score of 0 ~ 6), moderate (score of 7 ~ 12), and severe (score of 13 ~ 18). ADL consisted of nine items: bathing, dressing, grooming, mouth care, walking, climbing stairs, eating, transferring bed or chair, and getting toilet hygiene. The severity of ADL was determined as follows. Patients who gained a total score of 0 to 9 were considered to have mild symptoms. Those with a total score of 10 to 18 and 19 to 27 were defined as having moderate and severe symptoms, respectively. IADL included seven areas: ability to use a telephone, laundry, shopping, food preparation, housekeeping, taking medication, and handling finances. The severity of IADL was classified into three categories: mild (score of 0–7), moderate (score of 8–14), and severe (score of 15–21).

### Caregiver burden

Korea version of Zarit Burden Interview (ZBH-K) was used to measure caregiver burden [21]. Trained nurses were educated about burden interviews in advance. They recorded responses of caregivers during a 1:1 interview. Assessment tool was comprised of 22 domains, ranging from a score of 0 (not at all) to a score of 4 (have always been).

### Statistical analysis

Categorical data are presented as numbers and percentages. Continuous variables are presented as means and standard deviations. A chi-squared test was used to test the relationship between categorical variables. Multiple linear regression was used to analyze the relationship between CDR and characteristics of patients and caregivers. Patients’ CDR was used as a dependent variable which was stratified by severity level of dementia symptoms. All variables included in the bivariate analysis were entered in the multiple linear analysis except for patients’ and caregivers’ marital status and caregivers’ educational level. The marital status of the patients had strong collinearity with residence type. Therefore, we replaced marital status to residence type as one of the entry variables to understand the relationship between caregiver and patient. The reason for not including caregivers’ educational level is because more than 2000 caregivers did not answer for their education experience. Additionally, when we added the caregivers’ educational level to the regression model, no level of education was statistically significant, and the regression model was not improved. The number of missing in both patients’ and caregivers’ marital status were not large as that of caregivers’ educational level. However, it was not included in the model because of the same reason with the caregivers’ educational level.

SPSS version 20.0. (SPSS Inc., Chicago, IL, USA) was used for all statistical analyses. The probability level indicating statistical significance was set at *p* <  0.05.

## Results

### Characteristics of patients with dementia

Table 1 shows general characteristics of patients with dementia. The average age of patients was 80.5 years. The majority (68.4%) of them were females, which was twice as many as males (31.6%). The highest percentage was found for those with at least elementary education (36.9%) regarding education level, those who were bereaved (53.8%) regarding marriage status, those who were co-residing with other family members (36.9%) regarding co-resident type, and those who had no religion (36.5%) regarding religion status. Among patients classified by the severity of dementia, most patients had at least CDR 1 level (70.6%), followed by those with CDR 2 (18.5%) and CDR 3 (10.9%).

**Table 1:** General characteristics of patients with dementia, n (%)

Variables/Category		Original (*N* = 51,908)	Excluded (*N* = 39,616)	Included (*N* = 12,292)	χ^2^	*p*
Gender	Male	15,813 (30.5)	11,933 (30.1)	3880 (31.6)	9.229	0.0024
	Female	36,095 (69.5)	27,863 (69.9)	8412 (68.4)		
Age (Year)	< 60	629 (1.2)	539 (1.4)	90 (0.7)	90.155	< 0.0001
	60 ~ 69	4395 (8.5)	3497 (8.8)	898 (7.3)		
	70 ~ 79	18,137 (34.9)	13,872 (35.0)	4265 (34.7)		
	≥80	28,740 (55.4)	21,701 (54.8)	7039 (57.3)		
Educational level	Less than elementary education	17,369 (33.5)	13,567 (34.2)	3802 (30.9)	51.266	< 0.0001
	Elementary	18,411 (35.5)	13,880 (35.0)	4531 (36.9)		
	Junior High	5755 (11.1)	4336 (10.9)	1419 (11.5)		
	Senior High	6227 (12.0)	4691 (11.8)	1536 (12.5)		
	More than college education	4091 (7.9)	3087 (7.8)	1004 (8.2)		
CDR	Mild (0.5 ~ 1)	29,016 (67.6)	20,336 (51.3)	8680 (70.6)	1595.655	< 0.0001
	Moderate (2)	8004 (18.6)	5727 (14.5)	2277 (18.5)		
	Severe (≥3)	5931 (13.8)	4596 (11.6)	1335 (10.9)		
Residence type	Alone	10,494 (20.4)	8296 (20.9)	2198 (17.9)	334.746	< 0.0001
	With spouse	11,928 (23.2)	8721 (22.0)	3207 (26.1)		
	With spouse and other family members	7624 (14.8)	5704 (14.4)	1920 (15.6)		
	With other family members but no spouse	18,122 (35.2)	13,588 (34.3)	4534 (36.9)		
	Other (with friends or volunteer workers)	3280 (6.4)	2847 (7.2)	433 (3.5)		
Religion	Christian	14,964 (29.7)	11,082 (28.0)	3882 (31.6)	122.874	< 0.0001
	Buddhism	8257 (16.4)	6034 (15.2)	2223 (18.1)		
	Catholic	5785 (11.5)	4303 (10.9)	1482 (12.1)		
	No religion	20,447 (40.5)	15,956 (40.3)	4491 (36.5)		
	Confucianism	996 (2.0)	782 (2.0)	214 (1.7)		
Marital Status (*n* = 12,274)	Married	20,268 (39.4)	15,014 (37.9)	5254 (42.7)	147.608	< 0.0001
	Bereaved	28,674 (55.8)	22,057 (55.7)	6617 (53.8)		
	Separated	548 (1.1)	449 (1.1)	99 (0.8)		
	Divorced	950 (1.8)	816 (2.1)	134 (1.1)		
	Single	485 (0.9)	385 (1.0)	100 (0.8)		
	Cohabitated	505 (1.0)	435 (1.1)	70 (0.6)		

Note. *CDR* Clinical Dementia Rating.

### Results of SDAP

Results of SDAP are shown in Table 2. Most patients showed a moderate level of cognitive impairment (49.4%), behavior problems (82.6%), and ADL dependency (73.6%). However, a severe level of IADL dependency was the most frequent case (40.5%) among patients.

**Table 2:** Distribution of Seoul Dementia Assessment Packet (SDAP)

Variables	Category	n (%)	Mean ± SD^a^
Cognitive impairment	Mild (0–4)	4112 (33.5)	6.12 ± 2.64
	Moderate (5–8)	6076 (49.4)	
	Severe (9–12)	2104 (17.1)	
BPSD	Mild (0–6)	10,151 (82.6)	3.47 ± 3.56
	Moderate (7–12)	1791 (14.6)	
	Severe (13–18)	350 (2.8)	
ADL	Mild (0–9)	9051 (73.6)	6.26 ± 7.47
	Moderate (10–18)	2064 (16.8)	
	Severe (19–27)	1177 (9.6)	
IADL	Mild (0–7)	4122 (33.5)	11.91 ± 7.15
	Moderate (8–14)	3186 (25.9)	
	Severe (15–21)	4984 (40.5)	

^a^ *SD* Standard Deviation

*BPSD* Behavioral and Psychological Symptoms in Dementia, *CDR* Clinical Dementia Rating, *ADL* Activities of Daily Living, *IADL* Instrumental Activities of Daily Living

### Characteristics of caregivers

As shown in Table 3, most (68.5%) caregivers were females with an average age in the early 40s to late 50s (49.9%). They had 10 years to 12 years of upper secondary education (27.9%), co-residing with patients (63.8%), non-religion (45.8%), married (80.5%), unemployed (56.3%), and a moderate level of self-rated health status (56.0%). Mostly daughters were taking care of patients (27.8%) with no secondary caregiver (57.5%).

**Table 3:** Characteristics of Caregivers

Variables	Category	n (%) (*n* = 12,292)	Mean ± SD^a^
Gender	Male	3877 (31.5)	
	Female	8415 (68.5)	
Age (year)	< 40	790 (6.4)	58.67 ± 13.76
	40 ~ 49	2390 (19.4)	
	50 ~ 59	3754 (30.5)	
	60 ~ 69	2250 (18.3)	
	70 ~ 79	2160 (17.6)	
	> 80	948 (7.7)	
Educational level (*n* = 9990)	Less than elementary education	901 (7.3)	
	Elementary	1849 (15.0)	
	Junior High	1091 (8.9)	
	Senior High	3427 (27.9)	
	More than college education	2722 (22.1)	
Residence Type	Residing with patient	7844 (63.8)	
	Residing separately with patient	4448 (36.2)	
Religion	Christian	3564 (29.0)	
	Buddhism	1596 (13.0)	
	Catholic	1339 (10.9)	
	No religion	5635 (45.8)	
	Confucianism	158 (1.2)	
Marital Status (*n* = 12,138)	Married	9898 (80.5)	
	Bereaved	466 (3.8)	
	Divorced	447 (3.6)	
	Other (Separated, Cohabitating, or Single)	1327 (10.8)	
Relationship with patient	Spouse	3185 (25.9)	
	Daughter	3385 (27.5)	
	Son	2536 (20.6)	
	Other (Friends, volunteer worker)	3186 (26.0)	
Self-rated health status	Very bad	252 (2.1)	
	Bad	2014 (16.4)	
	Moderate	6884 (56.0)	
	Good	2761 (22.5)	
	Very Good	381 (3.1)	
Employment status	Currently working	5372 (43.7)	
	Unemployed	6920 (56.3)	
Receiving assistance for patient caretaking	Working with secondary caregivers	5229 (42.5)	
	Working independently	7063 (57.5)	

^a^ *SD* Standard Deviation

### Determinants of caregiver burden

General characteristics of patients with dementia and caregivers were used as independent variables in multiple regression analysis to identify significant factors affecting caregiver burden (Table 4). Before stratifying the severity of dementia, significant determinants of patients and caregivers were as follows. Male patients (β = − 1.396), and living with spouse and other family members (β = − 1.487) or others such as friends or volunteer workers (β = − 6.889) showed negative relationships with caregiver burden. Caregivers who were not co-residing with patients (β = − 3.769), who had Christian religion (β = − 1.071), who had no family relationship with patients (β = − 1.795), who answered that their recent health status was more than moderate (moderate: β = − 7.019, good: β = − 10.299), and who shared their works with secondary caregivers (β = − 2.399) showed similar results. However, patients with cognitive impairment (β = 1.312), limited ADL (β = 0.072), limited IADL (β = 0.698), and severe BPSD (β = 1.545) had positive relationships with caregiver burden. In the case of caregiver characteristics, those who were females (β = 3.386) or having parent and child relationships (daughter: β = 1.838, son: β = 2.835) had more caregiver burden.

**Table 4:** Caregiver burden according to characteristics of patients and caregivers

	All		CDR^a^					
			Mild (0.5~1)		Moderate (2)		Severe (3~5)	
	β	95% CI^b^	β	95% CI ^b^	β	95% CI ^b^	β	95% CI ^b^
**Patients with dementia**								
Sex (ref=female)	-1.396	-2.379,-0.412	-1.352	-2.512,-0.193	-1.529	-12.568,9.509	-0.198	-3.280,2.884
Age	-0.035	-0.088,0.017	-0.036	-0.101,0.029	-0.023	-2.393,2.347	-0.035	-0.174,0.103
Educational level (ref: less than elementary education)								
Elementary	0.654	-0.170,1.479	0.705	-0.288,1.697	0.289	0.168,0.411	0.178	-2.214,2.570
Junior High	-0.232	-1.439,0.975	0.345	-1.034,1.724	-1.965	-3.861,-0.068	-2.023	-6.470,2.424
Senior High	0.159	-1.026,1.345	-0.119	-1.481,1.243	1.081	-2.003,4.166	0.796	-3.273,4.864
More than college education	0.890	-0.548,2.327	1.740	0.107,3.373	-3.355	-6.390,-0.320	2.209	-3.182,7.599
CDR (ref: 0.5~1)								
2	0.485	-0.475,1.444						
>3	-1.244	-2.587,0.098						
Co-resident type (ref=Alone)								
With spouse	-0.694	-1.940,0.552	-0.908	-2.322,0.506	-0.064	-3.755,3.627	1.593	-2.870,6.055
With spouse and other family members	-1.487	-2.796,-0.179	-1.805	-3.299,-0.310	0.668	-2.577,3.912	-1.088	-5.580,3.404
With other family members but no spouse	0.072	-0.979,1.124	0.113	-1.135,1.362	0.806	-2.555,4.167	0.093	-3.017,3.202
Other (with friends or volunteer workers)	-6.889	-8.857,-4.921	-5.294	-8.551,-2.037	-6.451	-8.951,-3.951	-5.720	-9.395,-2.044
Religion (ref: no religion)								
Christian	0.737	-0.131, 1.605	0.502	-0.536,1.539	0.446	-3.559,4.451	1.671	-0.917,4.259
Buddhism	-0.121	-1.112, 0.870	-0.334	-1.486,0.817	0.821	-1.185,2.828	-1.706	-5.037,1.625
Catholic	-0.701	-1.924,0.522	-0.701	-2.137,0.734	0.467	-1.943,2.876	-2.963	-6.871,0.945
Confucianism	0.380	-2.167,2.927	0.758	-2.240,3.756	-2.174	-5.129,0.780	-0.238	-6.652,6.176
Cognitive impairment	1.312	1.130,1.494	1.451	1.233,1.670	1.025	-6.226,8.277	0.806	0.300,1.311
BPSD	1.545	1.435,1.655	1.683	1.533,1.832	1.368	0.932,1.804	1.206	0.956,1.456
ADL	0.072	0.003,0.142	0.130	0.033,0.227	0.157	-0.071,0.384	0.124	-0.042,0.290
IADL	0.698	0.628,0.767	0.706	0.626,0.786	0.267	0.132,0.402	0.052	-0.313,0.416
**Caregiver**								
Sex (ref: female)	3.386	2.169,4.603	3.717	2.301,5.134	1.131	0.937,1.325	4.172	0.430,7.914
Age	-0.012	-0.047,0.022	-0.021	-0.063,0.022	0.005	-3.148,3.158	0.021	-0.070, 0.111
Residing separately with patient (ref=Residing with patient)	-3.769	-4.634,-2.904	-3.125	-4.159,-2.091	-4.706	-4.784,-4.628	-5.726	-8.311,-3.140
Religion (ref: no religion)								
Christian	-1.071	-1.920,-0.222	-1.112	-2.125,-0.099	-0.491	-2.524,1.542	-1.788	-4.242,0.666
Buddhism	-0.104	-1.195,0.987	-0.650	-1.920,0.620	0.370	-1.639,2.380	2.713	-0.894,6.319
Catholic	0.743	-0.502,1.987	0.109	-1.366,1.583	2.055	-0.561,4.670	2.040	-1.724,5.804
Confucianism	0.016	-2.926,2.957	-0.179	-3.778,3.420	1.368	-1.574,4.310	-3.781	-11.872,4.310
Relation with patient (ref: spouse)								
Daughter	1.838	0.292,3.385	1.135	-0.694,2.964	0.872	-5.751,7.495	4.020	-1.128,9.168
Son	2.835	1.106,4.563	2.408	0.350,4.466	-0.013	-3.800,3.774	3.443	-2.008,8.893
Other (friends or volunteer worker)	-1.795	-3.397,-0.194	-1.502	-3.395,0.391	-3.326	-7.633,0.982	-3.326	-8.530,1.878
Self-rated health status (ref: Very Bad ~Bad)								
Moderate	-7.019	-7.937,-6.102	-6.392	-7.475,-5.309	-7.452	-11.329,-3.575	-9.470	-12.320,-6.619
Good ~ Very good	-10.299	-11.376, -9.222	-10.048	8.783,11.312	-10.380	8.236,12.524	-9.924	-13.348,-6.499
Economic activity (ref: unemployed)	-0.096	-0.838,0.647	0.151	-0.732,1.033	-0.122	-2.678,2.435	-0.617	-2.956,1.722
Receiving assistance for patient caretaking (ref: working independently)	-2.399	-3.091,-1.707	-2.110	-2.927,-1.294	-3.460	-5.191,-1.728	-1.429	-3.537,0.680
**adjusted R**^**2**^	.334		.312		.225		. 265	

*Note.* ^a^*CDR* Clinical Dementia Ratin

^b^*CI* Confidence Interval.

After stratifying the severity of dementia, results of CDR 1 were similar to overall patients’ caregiver burden. However, in case of CDR 2, patients who were living with others (β = − 6.451), caregivers who were not cohabitating with patients (β = − 4.706), who had moderate (β = − 7.452) or good (β = − 10.380) health status, or those who were working with secondary caregivers (β = − 3.460) showed less caregiver burden. On the other hand, female caregivers (β = 1.131) or patients who had limited IADL (β = 0.267) and severe BPSD (β = 1.368) had positive relationships with caregiver burden. Results of CDR 3 showed that patients who were co-residing with others (β = − 5.720) and caregivers who answered their health status as moderate (β = − 9.470) or good (β = − 9.924), and who were living independently with patients (β = − 5.726) showed less caregiver burden. In contrast with this, patients who were experiencing cognitive impairment (β = 0.806) or severe BPSD (β = 1.206) and female caregivers (β = 4.172) showed more burden.

## Discussion

This study analyzed relationships between caregiver burden and characteristics of patients with dementia and caregivers. A total number of 12,292 patients were analyzed, which outnumbered previous studies from Korea (609) and other countries (732) [22–24]. In this study, female patients were twice as many as male patients [25–28] because the prevalence of dementia had a positive relationship with age. In addition, life expectancy was different by gender.

Results also showed that 70% of patients had mild severity, implying that patients with more than moderate severity were residing in a nursing home while patients with mild severity were using SCD as an outpatient service. It was found that 41% of patients were residing with a spouse or other family members while 37% of patients were bereaved but living with other extended families. These results suggest that cultural background such as a strong Confucianism in Korea can influence patients’ family to be a major informal caregiver and accelerate caregiver burden [29].

Patients with worse cognitive functions and IADL were heavily relying on their caregivers. About 26% of them needed assistance for most of their daily activities. This finding suggests that most patients need the help of caregivers to keep their daily living, such as preparing a meal, taking medications, and managing financial statements due to their lack of ability to do so [30].

While taking care of patients with dementia, caregiver burden might exacerbate according to characteristics of caregivers. Considering that the mean age of caregivers was in the 60s, elderly care by elderly baby boomers not only could aggravate caregiver burden, but also could cause socioeconomic concern due to extensive health service use and unmet need [31]. Among patients’ SDAP evaluation criteria, cognitive impairment and limited IADL had significant relationships with caregiver burden. In particular, caregiver burden increased when patients were experiencing severe BPSD. Therefore, it would be critical to apply BPSD intervention programs for efficient patient care, consistent with findings of previous studies [32–34].

In addition to characteristics of patients, caregiver burden was related to determinants of caregivers. Male caregivers and those who were not residing with patients had a relatively lower caregiver burden. These results suggest that caregivers may be overwhelmed by the overly long working time for supporting patients with dementia. Therefore, proper allocation of caregivers’ work should be considered as one of the measurements for solving caregiver burden issues. Governmental strategies, considering caregivers’ self-rated health status and their patients’ clinical symptoms, should be arranged to relieve the caregiver burden. Intensive social support and social networks for caregivers are essential for solving the caregiver burden [19]. In particular, differentiated policy supports such as patients’ cognitive or behavioral problem-focused coping strategies or caregivers’ emotion-focused programs depending on caregivers’ needs, rather than applying the same coping plans [35], should be implemented considering the severity of patients’ clinical symptoms and caregivers’ situational coping strategies for more effective interventions.

After stratifying the severity of dementia, significant factors related to caregiver burden were different for each level of severity. Caregivers’ gender was a significant factor determining caregiver burden among patients with mild and severe severity, but not for those with moderate dementia symptoms. In contrast with this, having a secondary caregiver was related to less burden of caregivers for patients with mild and moderate severity of dementia, but not for those with severe dementia. The reason for such results might be because most patients with more than moderate severity were bedridden that required intensive care most of the time. Therefore, it would be more efficient to organize and apply health promotion program for patients with dementia and their caregivers based on dementia severity [36].

Lastly, caregivers for patients with severe dementia symptoms experienced a higher caregiver burden from patients’ limited cognitive function, problematic behavior, and caregivers’ negative health status. This implies the importance of supporting the health of caregivers for patients with severe symptoms of dementia, suggesting both physical and psychological health intervention programs for managing caregivers’ health status are needed [22, 37].

This study had some limitations. Firstly, most study participants were home-based patients recruited from an online database of SDMP. In addition, most (around 70%) study participants had mild symptoms. Thus, general characteristics of total patients might reflect traits of patients with mild severity. Therefore, results of this study could be only applied to a limited range of patients with dementia. Secondly, although a trained nurse had taken a series of training courses to measure caregiver burden, there might be observer variations. Also, some possible factors such as hours of caregiving, caregivers’ self-efficacy, and type of coping strategies could be included in this study due to limited information. Additionally, this study excluded participants who have no caregiver, CDR, or SDAP information or CDR scored 0. Considering the potential significance of their characteristics, the results of this study should be carefully interpreted. Nevertheless, this study had a strength in that it analyzed the relationship between caregiver burden and possible determinants considering both characteristics of patients with dementia and their caregivers in Korea with a large sample size. In particular, this study emphasized the importance of caring for the elderly since the elderly would become a grave social burden issue in the geriatric public health sector.

## Conclusion

This study analyzed the relationship between caregiver burden and socio-demographical characteristics of patients with dementia and caregivers by the severity of dementia symptoms. Results of analysis of 12,292 individuals enrolled in the Seoul Dementia Management Projects from 2010 to 2016 showed that gender was a significant factor affecting the burden of caregivers for patients with moderate or severe dementia symptoms. Additionally, secondary caregivers’ assistance was related to the burden of caregivers for patients with mild to moderate symptoms of dementia. However, caregivers’ self-rated health status and co-residence with patients also showed significant relationships with burden of caregiver for patients with severe symptoms of dementia.

This study demonstrated that caregivers taking care of patients with dementia experienced different levels of caregiver burden according to their socio-demographical characteristics and patients’ clinical and socio-demographical characteristics. In particular, caregivers’ health should be considered to prevent caregiver burden. Therefore, social supports with multiple coping strategies focusing on different levels of patients’ clinical symptoms and caregivers’ needs should be given. Governmental supports such as expanding beneficiaries for caregivers’ health management programs or providing secondary caregivers for mitigating caregivers’ workload would be essential.

## Data Availability

The data that support the findings of this study are available from Seoul Metropolitan Center for Dementia. However, restrictions may apply to the availability of these data, which were used under license for the current study, as they are not publicly available. Data are however available from the authors upon reasonable request and with permission of Seoul Metropolitan Center for Dementia.

## Abbreviations

NID: The National Institute of Dementia
SDMP: Seoul Dementia Management Project
CDR: Clinical Dementia Rating
SDAP: Seoul Dementia Assessment Packet
BPSD: Behavioral and Psychological Symptoms in Dementia
ADL: Activities of Daily Living
IADL: Instrumental Activities of Daily Living
ZBH-K: Korean version of Zarit Burden Interview
